# Quality of life and caregiver burden in pediatric glaucoma: A systematic review

**DOI:** 10.1371/journal.pone.0276881

**Published:** 2022-10-26

**Authors:** Amy Basilious, Steven Villani, Hyunsoo Jang, Karina M. Kaberi, Monali S. Malvankar-Mehta

**Affiliations:** 1 Department of Ophthalmology, Ivey Eye Institute, St. Joseph’s Health Care, London, Ontario, Canada; 2 Schulich School of Medicine and Dentistry, Western University, London, Ontario, Canada; 3 Department of Biology and The Biotron, Western University, London, Ontario, Canada; 4 Faculty of Nursing, School of Health Sciences, Western University, London, Ontario, Canada; 5 Department of Epidemiology and Biostatistics, Schulich School of Medicine and Dentistry, Western University, London, Ontario, Canada; World Health Organisation: Organisation mondiale de la Sante, INDIA

## Abstract

Pediatric conditions can lead to significant caregiver burden and poor quality of life (QoL). This systematic review describes research relating to caregiver burden and QoL of caregivers of pediatric glaucoma patients. A systematic database search of Embase, Medline, PsycINFO, CINAHL, Web of Science, and the three journals within the Association for Research in Vision and Ophthalmology (ARVO) was conducted in October 2021. Publications underwent abstract and full-text screening and were included if they reported pediatric caregivers’ QoL using quantitative or qualitative methods. Review articles, publications not in English, and articles focusing on adult glaucoma patients were excluded. Studies then underwent risk of bias assessment and data extraction. Of the 105 publications identified, 8 publications with 667 participants were included in the review. Studies indicated significantly higher burden and poor QoL in caregivers. Female sex, lower education level, lower income, and working status of caregivers were associated with poorer QoL and greater burden. Additionally, more severe and longer duration of the child’s disease negatively impacted these measures of caregiver wellbeing. Additionally, studies found significant improvement in caregiver QoL after patients underwent surgery with combined trabeculotomy-trabeculectomy. In conclusion, few studies have investigated the impact of pediatric glaucoma on caregivers. This review of the existing studies found poor QoL and high levels of caregiver burden within this population. Given the lifelong nature of pediatric glaucoma, there is a need for further longitudinal research focusing on the caregivers of these pediatric patients. Long-term follow-up would allow for a greater understanding of how caregiver QoL changes over the course of the disease.

## Introduction

Pediatric glaucoma patients report health related quality of life (QoL) scores similar to children with severe congenital cardiac defects and children who have undergone liver transplants [1]. Management of pediatric glaucoma is important to preventing the disease’s progression to blindness. This may require regular follow-up appointments, eye examinations, use of eye drops, and surgical procedures. The most common form of pediatric glaucoma, congenital glaucoma, is often managed with surgery, with the average patient undergoing 4.3 surgical procedures [2]. In the United States, it is estimated that the cost associated with pediatric glaucoma per individual exceeds $21,000 USD each year for the first 4 years [3]. Numerous appointments, surgeries, financial strain, and uncertain outcomes put considerable stress on caregivers. However, attention is not always given to the burdens that caregivers experience as a result. Caregiver burden is defined as the physical, psychological, emotional, social and financial stresses that individuals experience due to providing care [4].

Caregiver burden of adult patients has been discussed in the literature, but caregivers of pediatric patients are commonly overlooked in this research [5,6]. The burden of caregiving for adults with chronic eye diseases has been well-documented [7–9]. Caregivers of patients with age-related macular degeneration, for example, have reported high levels of depression and productivity loss due to missed work and personal obligations [8,9]. Interestingly, a recent study found caregiver burden in adult glaucoma to be negligible when compared with other chronic diseases such as Parkinson’s Disease and multiple sclerosis [10]. Burden, however, did increase with worsening visual field loss. Additionally, most patients in the study did not require a caregiver, contrary to pediatric glaucoma patients [10]. Research involving caregivers of pediatric patients is essential because there are important differences in the care of pediatric and adult diseases. For example, the annual cost of care for pediatric glaucoma is 1100% of the cost for adult glaucoma [3]. Furthermore, managing caregiver burden is essential in pediatric conditions, as children are known to mirror their caregivers’ emotional state. Children with more anxious parents, for example, have higher levels of anxiety [11]. Additionally, caregiving for pediatric patients can disrupt marital or partner relationships, and may lead to family dysfunction and social and behavioural problems for the child and their siblings [12,13].

To our knowledge, this is the first systematic review to report on caregivers of pediatric glaucoma patients. The aim of this review is to summarize the current research on caregiver burden and QoL, and to identify factors that contribute to poor QoL.

## Methods

### Search strategy and design

This systematic review followed reporting guidelines from PRISMA, the Preferred Reporting Items for Systematic Reviews and Meta-Analyses (S1 Table) [14]. A systematic search was performed using the following databases: Embase, Medline, PsycINFO, Cumulative Index of Nursing and Allied Health Literature (CINAHL), Web of Science and the Association for Research in Vision and Ophthalmology (ARVO). A search strategy was developed using key words that were a combination of terms relating to glaucoma, caregivers, and different aspects of quality of life. The search strategy was adapted to each database’s syntax and searched until October 10, 2021. No additional limitations for methodology, countries, location, etc. were applied to the searches. See S2 Table for each database’s search strategy and number of results.

### Inclusion and exclusion criteria

Inclusion criteria were publications in English and articles relating to any aspect of burden and QoL of caregivers of pediatric glaucoma patients. The grey literature and abstracts with no full texts available were included. Review articles and papers that focused on adult glaucoma patients were excluded.

#### Article screening

**Fig 1:**
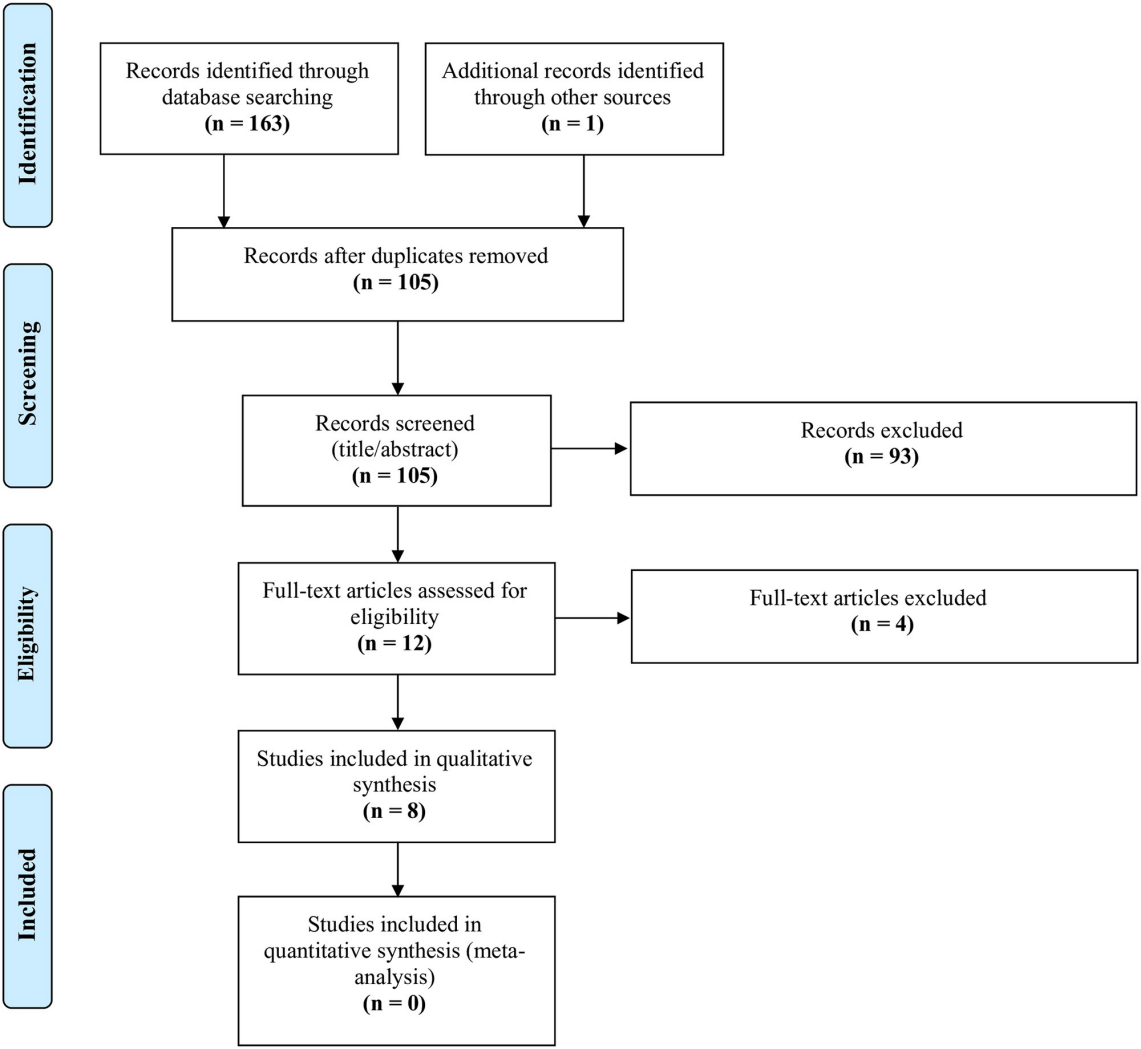
PRISMA flow diagram showing the inclusion and exclusion of studies.

Fig 1 outlines the PRISMA Flow diagram detailing article inclusion and exclusion. Covidence was utilized to detect duplicates and facilitate screening. First, title and abstracts of all search results were screened. Full text screening was then performed. Screening discrepancies between reviewers were noted and kappa statistics were calculated at each level of screening. Disagreements between reviewers were resolved through discussion and review of inclusion and exclusion criteria.

### Risk of bias

Two tools developed by the Clinical Advances Through Research and Information Translation (CLARITY) group at McMaster University were used to assess the risk of bias: the *Risk of Bias Instrument for Cross-Sectional Surveys of Attitudes and Practices* and the *Tool to Assess Risk of Bias in Longitudinal Symptom Research Studies Aimed at the General Population*. These tools provide questions that are scored 1 to 4, with higher scores indicating higher risk of bias. All publications were scored as low risk of bias. The risk of bias assessment is displayed in S3 and S4 Tables.

### Data extraction

Data extraction utilized a standardized data table which included the first author’s name, year of publication, country, aims of the study, study design, sample size, mean age, types of data gathered, data analysis methods, and main results of the studies. Study designs were cross-sectional surveys, retrospective chart reviews, or surveys before and after an intervention.

### Analysis

Due to the small number of heterogenous articles included, a meta-analysis could not be performed. Instead, a narrative synthesis was conducted.

## Results

### Search results

A search of each database was performed until October 10, 2021. The search yielded a total of 163 results, with 26 results from Embase, 76 from Medline, 2 from PsycINFO, 15 from CINAHL, 31 from Web of Science, and 13 from ARVO. One additional publication was identified through a reference search. Results were imported into Covidence, and duplicates were removed, yielding 105 articles. Title and abstract screening based on inclusion and exclusion criteria produced 12 articles, with a Cohen’s kappa statistic of 0.63. Full text screening resulted in 8 publications that met the criteria for the review, with a Cohen’s kappa statistic of 1. The results of the screening process are shown in Fig 1.

### Study characteristics

Of the 8 included studies, 6 were research articles and 2 were conference abstracts. All study participants presented to a single center in each study. Five publications were based on populations in India, one in Saudi Arabia, one in the USA, and one in China. Research interest in caregivers’ QoL is increasing, with the earliest two articles published in 2013, one each in 2014, 2016, and 2017, and three in 2019. Data extraction is summarized in Tables 1 and 2. All publications were scored as low risk of bias using the CLARITY group tools. The risk of bias assessment is displayed in S3 and S4 Tables.

**Table 1:** Study characteristics of the included articles.

Author & Year	Country	Study Design	Sample Size	Children’s age range
**AlQurashi et al. (2019) [21]**	Saudi Arabia	Cross-sectional Survey	46 fathers + 39 mothers	12.9 ± 3.8 years
**Dada et al. (2013) [22]**	India	Cross-sectional Survey	2 fathers + 53 mothers	8.1 ± 6.7 months
**Gothwal et al. (2014) [19]**	India	Before-After Interventional Survey	79 preoperative cohort and 63 postoperative cohort	6.1 ± 9.6 months
**Gothwal et al. (2016) [18]**	India	Before-After Interventional Survey	15 fathers + 96 mothers	5.7 ± 8.4 months
**Hanna et al. (2013) [23]**	USA	Retrospective Chart Review	60 patients, with 23 patients having data for 4 years	41.1 months
**Kantipuly et al. (2019) [17]**	India	Cross-sectional Survey	16 fathers + 54 mothers	7.7 ± 4.6 years
**Mandal et al. (2017) [20]**	India	Before-After Interventional Survey	5 fathers + 82 mothers	33.7 ± 8.8 months
**Zhu et al. (2019) [16]**	China	Cross-sectional Survey and Descriptive	19 fathers + 38 mothers	30.1 ± 35.1 months

**Table 2:** Quality of life scales used and outcomes of included studies. Abbreviations: CarCGQol (Caregiver’s Congenital Glaucoma Quality of Life), CBQ (Caregiver Burden Questionnaire), PHQ-9 (Patient Health Questionnaire), CBI (Caregiver Burden Index), PAC (Positive Aspects of Caregiving).

Author & Year	Data Measures	Data analysis	Main outcomes
**AlQurashi et al. (2019) [21]**	Caregiver’s Congenital Glaucoma Quality of Life (CarCGQoL) and demographic data	Rasch-calibrated scores and multiple linear analysis	Mean CarCGQoL score of caregivers was 0.63 (1.05). Poorer caregiver QoL was associated with: 1. Female gender (β = −0.52, 95% CI (−0.97, −0.06); p = 0.026) 2. Unemployment or retirement (β = −0.48, 95% CI (−0.96, −0.01); p = 0.044) 3. Caregiving for children with visual acuity of <20/200 in the better eye (β = −0.74, 95% CI (−1.23, −0.26); p = 0.003) 4. Having at least one other child with glaucoma (β = −0.92, 95% CI (−1.40, −0.43); p<0.001)
**Dada et al. (2013) [22]**	Caregiver Burden Questionnaire (CBQ), Patient Health Questionnaire (PHQ-9, which includes some demographic questions)	One-Way ANOVA	Aggregate caregiver burden was found to be moderate in 39 caregivers (71%) and severe in 3 caregivers (5%), with a mean CBQ score of 37.8 (10.9). Depression was present in 38 (69%) subjects, with 12 (22%) subjects having moderate depression and 6 (11%) having severe or very severe depression, with a mean PHQ-9 score of 8.90 (5.14). Burden scores increased with the severity of depression (p<0.0001).
**Gothwal et al. (2014) [19]**	CarCGQoL and PHQ-9, with basic demographic information	Rasch-calibrated scores, Cohen’s d (magnitude of change before and after surgery)	There was significant improvement in caregiver QoL and depression scores post-operatively (p<0.001), with Cohen’s d = 0.76 for CarCGQoL and Cohen’s d = 0.80 for PHQ-8. Demographic data and clinical variables did not predict the change in QoL.
**Gothwal et al. (2016) [18]**	CarCGQoL, overall QoL, and demographic data	Rasch-calibrated scores, effect size, Cohen’s d (magnitude of change before and after surgery)	Mean CarCGQoL score of caregivers was 0.84 (1.52) logits pre-operatively and 2.18 (1.90) logits postoperatively (mean difference = −1.34 logits; *t =* −6.82; p<0.0001), with Cohen’s d = −0.78 (95% CI: −0.50, −1.05). 71% of caregivers preoperatively and 20% of caregivers postoperatively reported their QoL to be “poor” or “very poor” (p<0.0001). Demographic data did not predict the change in QoL.
**Hanna et al. (2013) [23]**	Number of interventions performed per year	Trends	Burden (measured as number of interventions) was highest in the first year after diagnosis. On average, patients underwent 8.9, 5.7, 4.6, and 4.8 interventions in the first four years after diagnosis.
**Kantipuly et al. (2019) [17]**	PHQ-9, CarCGQoL, and demographic data. Grouping participants into high and low QoL groups for analysis	Rasch-calibrated scores, linear regression (univariate and multivariable analysis)	CarCGQoL was negatively associated with: 1. Child’s age (β = −0.04, 95% CI (−0.08, −0.01), p<0.05) 2. Child’s disease duration (β = −0.03, 95% CI (−0.07, −0.01), p<0.05) CarCGQoL and PHQ-9 scores were negatively associated (r = −0.66, p<0.01), with poorer QoL being correlated with higher depression scores.
**Mandal et al. (2017) [20]**	Socio-demographic data, clinical data, and QoL data	Rash-scaled scores, effect size	Improvement in caregiver’s QoL 2 years after their child’s glaucoma surgery (effect size = 1.07).
**Zhu et al. (2019) [16]**	Demographic data, Caregiver Burden Index (CBI), Positive Aspects of Caregiving (PAC)	Pearson correlation analysis, linear regression model	Caregivers had mild (23, 40.4%), moderate (29, 47.4%), and severe (5, 8.8%) levels of burden. Caregivers had mild (9, 15.8%), moderate (31, 54.4%), and severe (17, 29.8%) PAC scores. Greater burden scores were associated with: 1. Longer duration (>1 year) of the child’s disease (χ^2^(4, N = 57) = 10.92; p = 0.027) 2. Female gender (χ^2^(1, N = 57) = 6.20; p = 0.013), 3. Employment (χ^2^(1, N = 57) = 8.38; p = 0.004) 4. Education level (χ^2^(4, N = 57) = 11.49; p = 0.022) 5. Household income (χ^2^(4, N = 57) = 10.76; p = 0.03)

Four publications utilized a specialized survey that was developed by Gothwal et al. (2015) for measuring the QoL of caregivers of children with primary congenital glaucoma (CarCGQoL) [15]. Three other publications utilized different combinations of surveys for QoL such as the Caregiver Burden Questionnaire (CBQ), Patient Health Questionnaire (PHQ-9), Caregiver Burden Index (CBI), and Positive Aspects of Caregiving (PAC). The CBQ measures socioeconomic burden, psychological burden and burden associated with providing care. The PHQ-9 is a depression assessment. The CBI tests caregiver burden in 5 subscales: time dependence, developmental, behaviour, physical burden, social burden, and emotional burden. The PAC is a 10-item questionnaire measuring the positive aspects of caregiving. One publication utilized the number of interventions as a proxy for burden on caregivers. Four of the studies were cross-sectional surveys, 3 performed surveys before and after glaucoma surgery, and one was a retrospective chart review. Seven of the 8 publications collected sociodemographic data.

### Caregiver burden and quality of life

Zhu et al. (2019) found that caregivers of pediatric glaucoma patients were found to have mild or moderate levels of burden, measured by the CBI questionnaire [16]. Caregiver stress is multifaceted, stemming from concerns beyond caregiving. Kantipuly et al. (2019) noted that approximately 70% of caregivers worried ‘very much’ about the child’s vision after surgery and the child’s marriage prospects [17]. In the same study, the PHQ-9 was utilized to assess symptoms of depression. They found that 44% of respondents had varying degrees of depressive symptoms. However, they noted that the PHQ-9 was poorly suited culturally to the study population.

Gothwal et al. (2016) found that all caregivers endorsed items on the CarCGQoL questionnaire relating to burden, with 60% responding with “very much” or “moderate amount” [18]. Six to eight weeks postoperatively, 55% of caregivers chose “not at all” for the same CarCGQoL items. The mean CarCGQoL score significantly improved from 0.84 ± 1.52 logits preoperatively to 2.18 ± 1.90 logits postoperatively [18]. Gothwal et al. (2014) found a statistically significant improvement in CarCGQoL and PHQ-9 scores six weeks postoperatively (Cohen’s d = 0.76 for QoL and 0.80 for depression) [19]. Mandal et al. (2017) measured caregivers’ CarCGQoL preoperatively, 8 weeks postoperatively, and 2 years postoperatively [20]. The mean scores at those timepoints were -0.09 ± 1.30, 0.88 ± 1.60, and 2.13 ± 2.29, demonstrating that QoL scores increased 2 months and 2 years postoperatively. The large increases between pre and postoperative quality of life suggests that treatment of a child’s glaucoma not only improves disease outcomes but also improves caregiver QoL.

AlQurashi et al. (2019) found poor QoL for parents, with a mean CarCGQoL score of 0.63 ± 1.05 [21]. Interestingly, this cross-sectional survey reported no significant correlation between QoL scores and number of surgical interventions [21]. Dada et al. (2013) surveyed caregivers of post-operative pediatric glaucoma patients [22]. They found that all caregivers experienced some degree of burden, with 39 (71%) having moderate aggregate burden and 3 (5%) having severe aggregate burden measured by the CBQ. In addition, 38 (69%) caregivers had depression, with 12 (22%) having moderate depression and 6 (11%) having severe depression as measured by the PHQ-9.

Despite longitudinal studies showing that QoL increases significantly postoperatively [18–20], some cross-sectional studies indicate that the number of interventions a child has had is not associated with their caregiver’s quality of life [17,21,22]. Hanna et al. (2013) found that the average number of interventions in the first 4 years of diagnosis was 8.9, 5.7, 4.6, and 4.8 respectively [23]. The total direct cost over the first four years was estimated to be approximately $189,275 USD by Huang et al. (2014) [24]. The frequency of procedures and cost of procedures could be more burdensome for caregivers with lower socioeconomic status. However, different healthcare systems create varying levels of burden on caregivers depending on costs associated with procedures. For example, the cross-sectional survey from Saudi Arabia conducted by AlQurashi et al. (2019) found no significant association between the number of surgical procedures and QoL [21].

### Demographic factors

Several studies found associations with sociodemographic traits. AlQurashi et al. (2019), in a bivariate linear regression analysis, found that caregivers with significantly worse CarCGQoL scores were female (β = −0.52, 95% CI (−0.97, −0.06); p = 0.026), unemployed or retired (β = −0.48, 95% CI (−0.96,−0.01); p = 0.044), caregivers for children with visual acuity of <20/200 in the better eye (β = −0.74, 95% CI (−1.23,−0.26); p = 0.003), and have at least one other child with glaucoma (β = −0.92, 95% CI (−1.40, −0.43); p<0.001) [21]. Having other children affected by glaucoma was the strongest correlate of poor QoL. The authors noted that mothers may be more likely to provide more care to the affected child or children, therefore incurring greater levels of burden and poorer QoL scores. Results from Kantipuly et al. (2019) found that CarCGQoL scores were significantly negatively associated with the child’s age (β = −0.04, 95% CI (−0.08, −0.01); p<0.05) and duration of disease (β = −0.03, 95% CI (−0.07, −0.01); p<0.05) [17].

Zhu et al. (2019) found no correlation between demographic data and PAC scores [16]. There were, however, correlations between demographic data and CBI scores. CBI scores indicated greater burden for caregivers who were female (χ^2^(1, N = 57) = 6.20; p = 0.013), employed (χ^2^(1, N = 57) = 8.38; p = 0.004), caregivers to patients with a disease duration greater than one year (χ^2^(4, N = 57) = 10.92; p = 0.027), and who had lower education levels (χ^2^(4, N = 57) = 11.49; p = 0.022) and lower household income (χ^2^(4, N = 57) = 10.76; p = 0.03).

Studies measuring QoL before and after a surgical intervention found that sociodemographic characteristics did not predict the change in QoL. Caregiver QoL significantly increased postoperatively regardless of the demographic and clinical variables of the caregivers and patients [18,19]. Therefore, successful surgery, irrespective of demographic factors, improves the caregiver’s QoL postoperatively.

## Discussion

Pediatric glaucoma is a lifelong condition requiring intensive resources by the caregivers. The negative impacts of caregiving for a child with glaucoma emphasizes the need for wholistic management. This study is the first systematic review to report on caregiver burden and QoL in pediatric glaucoma. Caregiving for this complex disease presents significant emotional, social, and financial challenges. Aggregate results from this review indicate that the caregiver’s female gender, employment status, and longer duration of disease reduce caregivers’ QoL and increase caregiver burden [16,17,21]. Individual studies have also found that lower level of education, lower household income, caregiving for other children with glaucoma, and the child’s older age and poorer visual acuity adversely impact these measures of caregiver wellbeing [16,17,21]. Lower levels of education may make it more challenging for caregivers to learn about the disease and management plan [16]. Longer duration of treatment and poorer visual acuity may indicate worse disease and a greater need for support [16,17]. Previous research has similarly found sociodemographic and psychosocial factors to predict up to 40% of the variance in caregiver burden and QoL in chronic pediatric conditions [25,26]. In particular, higher monthly family income, more social supports, and greater parental resilience (defined as strength and self-confidence, social competence, family and social support) were factors that predicted better quality of life [26]. Family functioning, measured by the domains of positive family environment, cohesion, hostility/conflict avoidance, and rules/emotional expression, was also an important contributor to better QoL.

Furthermore, the studies in our review found higher levels of burden and lower levels of QoL were correlated with greater depressive symptoms [22]. Similarly, previous research has shown that caregivers’ psychosocial factors impact burden, with greater levels of depression predicting poorer QoL, and anxiety predicting greater caregiver burden [26]. Poor caregiver mental health has even been found to predict worse patient outcomes [27]. It is important for healthcare providers to be cognizant of caregiver burden and consider referral to community supports, mental health professionals, and/or virtual support groups.

Studies utilizing questionnaires before and after surgical intervention with combined trabeculotomy-trabeculectomy supported an increase in caregivers’ QoL after the child’s surgery [18–20]. Therefore, the impacts of interventions on caregivers may be considered as well. This improvement may reflect hope that surgery will help improve the course of their child’s disease. Surgery may be viewed as an intervention against the vison loss that may occur in pediatric glaucoma. Furthermore, IOP lowering is a very tangible result of surgery that may be encouraging for caregivers. However, studies with a cross-sectional survey completed after surgery still report low caregiver QoL [21,22]. These cross-sectional studies do not reflect the changes in caregiver QoL that occur at different time intervals post-operatively. Research with caregivers of pediatric orthopedic patients has shown that caregiver stress increases immediately post-operatively, with levels decreasing one year later [28]. In the context of eye surgery, post-operative care may be especially challenging in pediatric patients. They may require more frequent eyedrops and a shield to protect the eye, which children may resist. Furthermore, post-operative follow-up visits may add to the burden.

The studies evaluated in this literature review reported varying burden and depression levels of caregivers. Our study was limited by the heterogeneity between studies, preventing us from conducting a meta-analysis. Factors such as differences in study designs, variations in questionnaires used, and variable follow-up periods prevented a quantitative synthesis. However, this heterogeneity also indicates that the findings are robust, as different measures and study designs reported similar results. Furthermore, our systematic review was limited by the small number of publications that have studied the QoL of caregivers of pediatric glaucoma patients. Importantly, no studies assessed the impact of family and social supports or disease education on QoL, which research has shown to be protective factors for pediatric caregiver wellbeing [26]. These factors should be explored in future research, as they may represent targets for caregiver burden interventions.

Further studies are also necessary to elucidate how different societies and cultures may experience caregiver burden. The utilization of standardized scales to measure QoL amongst future studies would provide direct and comparable data. Additionally, study designs using matched cohorts of similar sociodemographic background with repeat QoL assessments over time will provide better insight into the characteristics that affect QoL.

## Conclusion

Current research suggests that caregivers of pediatric glaucoma patients experience poor QoL. In addition, certain sociodemographic characteristics such as a caregiver’s female gender, employment status, household income, and education level are associated with poorer QoL and greater burden. Duration and severity of the child’s disease and additional caregiving burdens further contribute. Successful surgical treatment of pediatric glaucoma results in an improvement in caregivers’ QoL postoperatively. Future research should study QoL changes over time and investigate the role of disease education for caregivers and financial governmental supports in improving caregiver QoL.

## Supporting information

S1 Table PRISMA guidelines checklist. (DOCX)

S2 Table Search strategies for each database and number of results until October 10, 2021. (DOCX)

S3 Table Results of risk of bias assessment using the risk of bias instrument for cross-sectional surveys of attitudes and practices. Scored 1–4 with higher scores indicating higher risk of bias.
(DOCX)

S4 Table Results of risk of bias assessment using the tool to assess risk of bias in longitudinal symptom research studies aimed at the general population. Scored 1–4 with higher scores indicating higher risk of bias.
(DOCX)
