# Supplementary material for: Quality of life and caregiver burden in pediatric glaucoma: A systematic review
Source: PLoS One. 2022 Oct 26;17(10):e0276881. doi: 10.1371/journal.pone.0276881 (PMC9605022; doi:10.1371/journal.pone.0276881)
Supplement: S2 Table — (DOCX) [file pone.0276881.s002.docx]

**Table S2.** Search strategies for each database and number of results until October 10, 2021.

| Database | Search Strategy | Search Results |
| --- | --- | --- |
| Ovid Medline ALL (Ovid) | ((strain* or burden* or QOL* or quality of life* or quality-of-life* or depression* or anxiety* or disability* or mental health* or impact or well-being* or well being* or satisfaction*) and (caregiver* or care giver* or care-giver* or caretaker* or care-taker* or care taker*) and glaucoma*).mp. | 26 |
| Embase Classic + Embase | ((strain* or burden* or QOL* or quality of life* or quality-of-life* or depression* or anxiety* or disability* or mental health* or impact or well-being* or well being* or satisfaction*) and (caregiver* or care giver* or care-giver* or caretaker* or care-taker* or care taker*) and glaucoma*).mp. | 76 |
| PsycInfo (proquest) | ((strain* or burden* or QOL* or quality of life* or quality-of-life* or depression* or anxiety* or disability* or mental health* or impact or well-being* or well being* or satisfaction*) and (caregiver* or care giver* or care-giver* or caretaker* or care-taker* or care taker*) and glaucoma*) | 2 |
| Cumulative Index of Nursing and Allied Health Literature (CINAHL) | ((strain* or burden* or QOL* or quality of life* or quality-of-life* or depression* or anxiety* or disability* or mental health* or impact or well-being* or well being* or satisfaction*) and (caregiver* or care giver* or care-giver* or caretaker* or care-taker* or care taker*) and glaucoma*)  Uncheck ‘suggested search terms’. | 15 |
| Web of Science | (((QOL or quality of life or quality-of-life or depression or anxiety or disability or mental health or impact or well-being or well being or satisfaction) and (caregiver* or care giver* or care-giver* or caretaker* or care-taker* or care taker*) and glaucoma*))  Searched ‘ALL FIELDS’ | 31 |
| Association for research in Vision and Ophthalmology (ARVO) | ((strain or burden or QOL or quality of life or quality-of-life or depression or anxiety or disability or mental health or impact or well-being or well being or satisfaction) and (caregiver or care giver or care-giver or caretaker or care-taker or care taker) and glaucoma) | 13 |
