# Supplementary material for: Quality of life and caregiver burden in pediatric glaucoma: A systematic review
Source: PLoS One. 2022 Oct 26;17(10):e0276881. doi: 10.1371/journal.pone.0276881 (PMC9605022; doi:10.1371/journal.pone.0276881)
Supplement: S3 Table — Scored 1–4 with higher scores indicating higher risk of bias. (DOCX) [file pone.0276881.s003.docx]

**Table S3.** Results of Risk of Bias assessment using the *Risk of Bias Instrument for Cross-Sectional Surveys of Attitudes and Practices*. Scored 1-4 with higher scores indicating higher risk of bias.

| Risk of Bias Questions | Publication (Author, Year) | | | | | | |
| --- | --- | --- | --- | --- | --- | --- | --- |
|  | Alqurashi, 2019 | Dada, 2013 | Gothwal, 2014 | Gothwal, 2016 | Kantipuly, 2019 | Mandal, 2017 | Zhu, 2019 |
| Is the source population representative of the population of interest? | 2 | 2 | 2 | 2 | 2 | 2 | 2 |
| Is the Response Rate Adequate? | 1 | 1 | 1 | 1 | 1 | 1 | 2 |
| Is there little missing data? | 1 | 1 | 1 | 1 | 1 | 2 | 1 |
| Is the survey clinically sensible? | 1 | 2 | 1 | 1 | 1 | 1 | 2 |
| Is there any evidence for the reliability and validity of the survey instrument? | 2 | 2 | 2 | 2 | 2 | 2 | 2 |
