# Supplementary material for: Quality of life and caregiver burden in pediatric glaucoma: A systematic review
Source: PLoS One. 2022 Oct 26;17(10):e0276881. doi: 10.1371/journal.pone.0276881 (PMC9605022; doi:10.1371/journal.pone.0276881)
Supplement: S4 Table — Scored 1–4 with higher scores indicating higher risk of bias. (DOCX) [file pone.0276881.s004.docx]

**Table S4.** Results of Risk of Bias assessment using the Tool to Assess *Risk of Bias in Longitudinal Symptom Research Studies Aimed at the General Population.* Scored 1-4 with higher scores indicating higher risk of bias.

| Risk of Bias Questions | Publication (Author, Year) |
| --- | --- |
|  | Hanna, 2013 |
| Is the source population (sampling frame) representative of the general population? | 2 |
| Is the assessment of the outcome accurate both at baseline and at follow-up? | 1 |
| Is there little missing data? | 1 |
